# Antibacterial effect of silver nanorings

**DOI:** 10.1186/s12866-020-01854-z

**Published:** 2020-06-19

**Authors:** Sara González-Fernández, Víctor Lozano-Iturbe, Beatriz García, Luis J. Andrés, Mª. Fe Menéndez, David Rodríguez, Fernando Vazquez, Carla Martín, Luis M. Quirós

**Affiliations:** 1 Instituto Universitario Fernández-Vega, Instituto de Investigación Sanitaria del Principado de Asturias, and Departamento de Biología Funcional, Universidad de Oviedo, 33006 Oviedo, Spain; 2 Departamento de Fotónica-ITMA Materials Technology, 33490 Avilés, Spain; 3 Instituto Universitario de Oncología del Principado de Asturias and Departamento de Bioquímica y Biología Molecular, Universidad de Oviedo, 33006 Oviedo, Spain; 4 Servicio de Microbiología, Hospital Universitario Central de Asturias, 33011 Oviedo, Spain

**Keywords:** Silver nanoparticles, Silver nanorings, Silver nanospheres, Silver nanowires, Antibacterials

## Abstract

**Background:**

The emergence and expansion of antibiotic resistance makes it necessary to have alternative anti-infective agents, among which silver nanoparticles (AgNPs) display especially interesting properties. AgNPs carry out their antibacterial action through various molecular mechanisms, and the magnitude of the observed effect is dependent on multiple, not fully understood, aspects, particle shape being one of the most important. In this article, we conduct a study of the antibacterial effect of a recently described type of AgNP: silver nanorings (AgNRs), making comparisons with other alternative types of AgNP synthesized in parallel using the same methodology.

**Results:**

When they act on planktonic forms, AgNRs produce a smaller effect on the viability of different bacteria than nanoparticles with other structures although their effect on growth is more intense over a longer period. When their action on biofilms is analyzed, AgNRs show a greater concentration-dependent effect. In both cases it was observed that the effect on inhibition depends on the microbial species, but not its Gram positive or negative nature. Growth patterns in silver-resistant *Salmonella* strains suggest that AgNRs work through different mechanisms to other AgNPs. The antibacterial effect is also produced to some extent by the conditioning of culture media or water by contact with AgNPs but, at least over short periods of time, this is not due to the release of Ag ions.

**Conclusions:**

AgNRs constitute a new type of AgNP, whose antibacterial properties depend on their shape, and is capable of acting efficiently on both planktonic bacteria and biofilms.

## Background

Nanoparticles (NPs) are structures characterized by being less than 100 nm in size. This gives them unique properties, which endows them with significant potential as transport, diagnostic or therapeutic agents in different biomedical applications [1, 2].

One of the fields where NPs are of particular interest is their use as anti-infective agents. The expansion of resistance to various antimicrobial drugs available, as well as to newly developed antibiotics, and the corresponding increase in infections acquired both in hospitals and in the community, makes the development of alternative treatments imperative. Silver has been reintroduced into the field of biomedicine as a promising strategy to counter this worrying trend, and AgNPs have been found to be a less toxic alternative than the use of silver ions [3–6].

The bactericidal capacity of AgNPs is influenced by their physical-chemical properties and, in particular, by factors such as concentration, size or shape, in addition to the biological characteristics of the microorganism on which they act [7, 8]. The concentration of AgNPs is known to be an essential factor in their toxic effect, as well as being dependent on the specific type of microorganism; Gram-positive bacteria show lower susceptibility to AgNPs than those that are Gram-negative, although both types experience complete inhibition of their growth at concentrations > 75 μg/ml [9]. The concentration-dependent antibacterial effect has also been found to be independent of the bacteria acquiring antibiotic resistance [10]. Another important parameter that conditions the bactericidal properties of AgNPs is size. For a given volume of AgNP, as particle size decreases the specific surface area increases, thus allowing a proportional increase in its interaction with the surrounding environment [4]. In general, therefore, the smaller nanoparticles appear to have a higher toxicity [11–13].

The manner of the interaction between AgNPs and microorganisms also depends on the shape of the former. It has been found that AgNPs with the same surface area, but with different shapes, show different antibacterial activities, and this has been related to variations in their effective surface and other aspects of their surface chemistry [8, 11, 12]. However, based on current knowledge, the physical-chemical basis of the influence of shape on the antibacterial activity of AgNPs remains unclear.

A review of several studies has described various types of mechanisms by which AgNPs are able to carry out their toxic effect [11]. When bacteria are exposed to AgNPs, the latter adhere to the bacterial membrane or cell wall, which can lead to wall rupture, morphology changes, increased membrane permeability and serious alterations in cell physiology [11, 14–16]. After adhesion, AgNPs are able to penetrate the cell, where they are able to interact with different cell structures and biomolecules, thus affecting cell viability [11]. On the other hand, a number of studies have linked the antibacterial activity of AgNPs to an increase in the oxidative stress of cells [11], although the mechanism is not fully clear [17]. It has also been suggested that AgNPs carry out their antibacterial effect through the modulation of signal transduction pathways capable of affecting growth and other cellular activities [11].

Both Gram positive and Gram negative microorganisms can form surface-associated communities, called biofilms. The bacteria in the biofilms have remarkably altered characteristics, such as an ability to resist antimicrobial agents, as well as being of great clinical importance since biofilms are responsible for numerous persistent infections [18]. Several studies have shown that AgNPs are also effective bactericidal agents for the treatment of biofilms [4], and, as with planktonic bacteria, their effect is dependent on factors such as concentration, size and shape, in addition to the specific bacterial species involved [19–21], although the precise mechanism by which AgNPs operate on biofilms is not yet very well understood [18].

In this article, we analyze for the first time the antibacterial activity of a form of recently described AgNP, silver nanorings (AgNRs) [22, 23], and compare it with that presented by other morphologies such as nanowires (AgNWs) and nanospheres (AgNSs), all of which were obtained using a common synthesis procedure. The study was carried out on various pathogens, both Gram positive and Gram negative, and on microorganisms in both planktonic form and in biofilms. Finally, a possible mechanism for the antibacterial action of the different morphologies of AgNPs is analyzed.

## Results

### The antibacterial effect of AgNPs depends on their structure and the bacteria with which they interact

The appropriate proportion of bacteria:AgNPs to be used in the experiments was determined using *E. coli* as a reference against various proportions of AgNPs. The results showed that an increase in the concentration of AgNPs had a progressive but limited effect on bacterial viability up to an AgNP:bacteria ratio of 10^− 2^:1, and then greatly increasing from this point and reaching mortality values around 93–95% at 1:1 ratios, with less pronounced increases at higher ratios. Since the comparison of the toxic effect of the different AgNPs could be best made at concentrations that did not involve total mortality, the 1:1 ratio was chosen as the most suitable for subsequent experiments (data not shown).

**Fig. 1:**
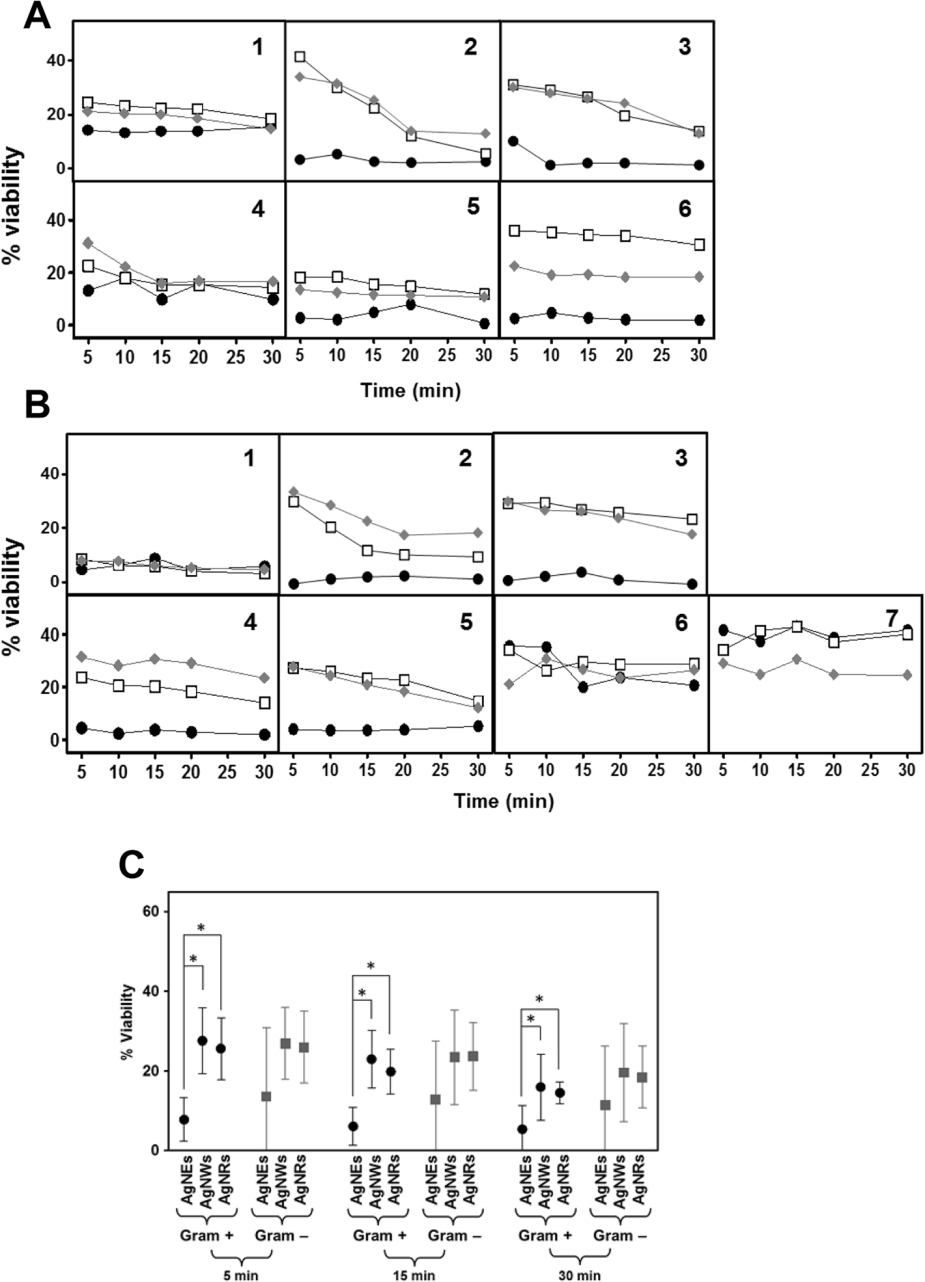
Antibacterial effect of AgNPs. **a** Effect of AgNPs on Gram positive bacteria: 1, *S.aureus*; 2, *S. epidermidis*; 3, *B. globisporus*; 4, *S. pneumoniae*; 5, *E. faecalis*; 6, *S. pyogenes*. **b** Effect of AgNPs on Gram negative bacteria: 1, *E. coli;*2, *H. influenzae*; 3*, K. pneumoniae*; 4, *S. typhimurium*; 5, *S. marcescens*; 6, *P. aeruginosa*; 7, *N. gonhorreae*. (●) AgNSs, (□) AgNWs, (♦) AgNRs. **c** Comparison of the effect produced by each specific type of nanostructure on the set of Gram positive bacteria versus the set of Gram negatives for different incubation times; statistically significant differences (*p* < 0.01) are indicated by asterisks

The study of the toxic effect of AgNPs on different bacterial species detected, in all cases, a high biocidal effect, although the observed patterns were variable depending on the microorganism tested and the type of AgNP used (Fig. 1). When Gram positive microorganisms were studied, in all cases AgNSs caused a bacterial mortality at least equal to, and in most cases higher than, that observed using the other structures. AgNWs and AgNRs produced very similar effects except in the case of *S. pyogenes*, where the toxicity of the AgNRs was higher. In general, AgNSs were able to produce their toxic effect after only 5 min of incubation, with no significant further alterations being observed when using longer incubation periods. AgNWs and AgNRs, however, produced an effect that was, in several cases, dependent on incubation time in that it progressively increased with increasing incubation time: an effect that was very evident in *S. epidermidis* and *B. globisporus* and, to a lesser extent, in *S. pneumoniae* (Fig. 1a). These results led to a significant negative correlation between bacterial survival values and incubation time, with R = − 082 for AgNWs and R = − 0.59 for AgNRs, but not for AgNSs.

When the tests were conducted using Gram-negative bacteria, the results, in part, were coincident with those obtained for the Gram-positives. Thereby, in most cases the AgNSs produced a greater observable effect at short times little subsequent change at longer times; likewise, AgNWs and AgNRs produced toxicity, although in many cases it was lower than that produced by AgNSs and, in addition, in the cases of *H. influenzae*, *K. pneumoniae*, *S. typhimurium* and *S. marcescens,* the effect of AgNWs and AgNRs was dependent on the incubation time (R = − 0.67 and R = − 0.77 respectively). The one exception to the trend was *E. coli*, where the effects observed were very similar for all three nanostructures and were independent of the incubation time. Another point of note is that for *P. aeruginosa* and *N. gonorrhoeae*, the observed toxicity was lower than for the other Gram negatives, even at longer incubation periods. In addition, in *N. gonorrhoeae* the largest toxic effect was produced by AgNRs (Fig. 1b).

A statistical analysis of the results obtained was carried out. In Gram positive bacteria, the existence of a statistically significant difference for AgNSs in comparison with both AgNWs and AgNRs was detected (*p* < 0.01 in both cases). Although the toxicity values obtained for the different nanostructures ​​became progressively more similar over time, this statistically significant difference was detected throughout the entire study (Fig. 1c). In contrast, in Gram negative bacteria, despite the greater effect produced by the AgNSs, no statistically significant differences were detected compared to the other AgNPs, which could be explained by the great variability in the effects of the AgNSs on the different bacterial species (Fig. 1c). In no case were any statistically significant differences detected for a specific nanostructure when comparing the results of the Gram positive and the Gram negative bacteria (Fig. 1c).

### Influence of AgNPs on bacterial growth depends on their morphology and the pathogen involved

**Fig. 2:**
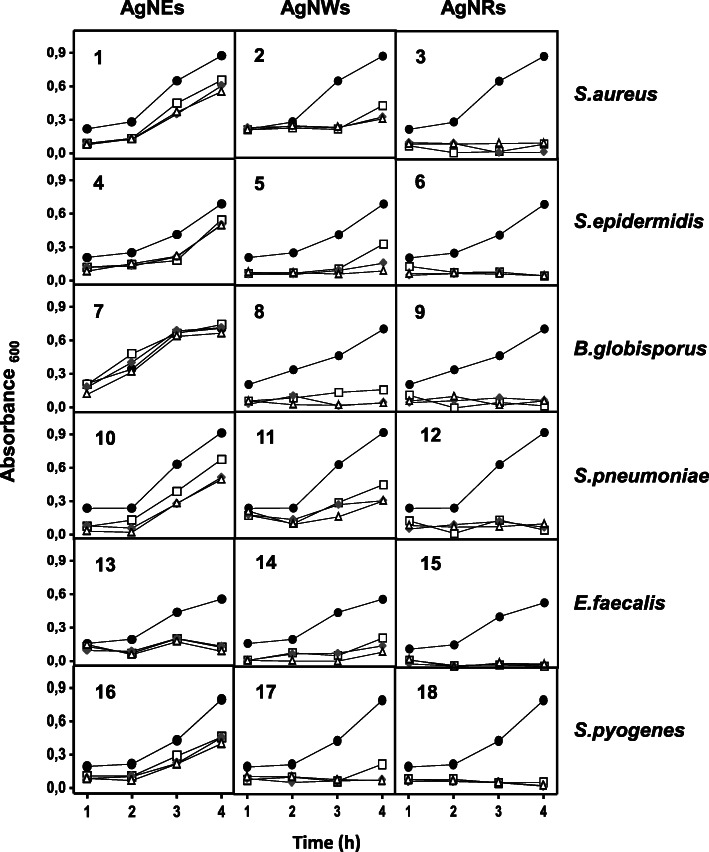
Influence of AgNPs on the growth of Gram positive bacteria. Ratios of bacteria:AgNPs used: (●) 1:0, (□) 1:1, (♦) 1:2, (∆) 1:3

**Fig. 3:**
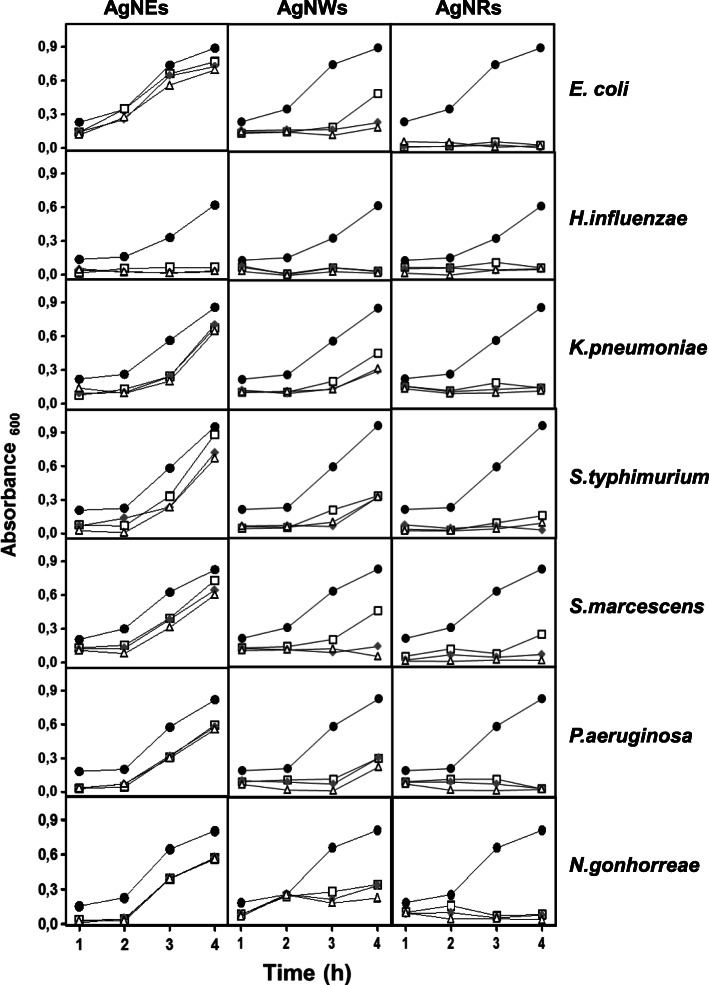
Influence of AgNPs on the growth of Gram negative bacteria. Ratios of bacteria:AgNPs used: (●) 1:0, (□) 1:1, (♦) 1:2, (∆) 1:3

The effect of AgNPs on growth curves was also analyzed. The results showed that AgNP influence was sometimes dependent on the specific bacterial species involved although general patterns related to the type of nanostructure used were discernable (Figs. 2 and 3). The AgNSs did not show a high capacity for bacterial growth inhibition in most of the microorganisms analyzed. The considerable inhibitory capacity of AgNWs, on the other hand, was evident in all the cases analyzed and AgNRs strongly inhibited bacterial growth in the long term in all cases (Figs. 2 and 3).

### The antibacterial effect of AgNPs on silver-resistant *S. enterica* shows distinctive strain-dependent patterns

**Fig. 4:**
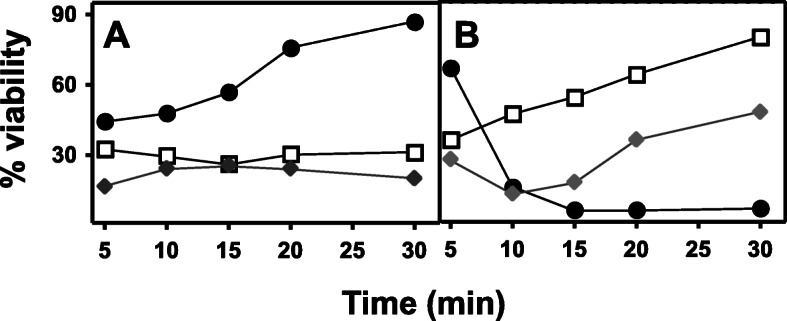
Effect of AgNPs on *S. enterica* strains resistant to silver. Antibacterial effect of AgNPs on: **a***S. enterica* serovar *typhimurium* 207/07; **b***S. enterica* serovar *typhimurium* 389/97. (●) AgNSs, (□) AgNWs, (♦) AgNRs

The availability of certain silver resistant strains of *Salmonella enterica* allowed us to determine the effect on them of different types of AgNPs as compared to the sensitive phenotype. The viability patterns obtained for these resistant strains after contact with AgNPs were different from the sensitive strain and, interestingly, also differed between the two strains. *S. enterica* 207/07 showed high resistance to AgNSs, while *S. enterica* 389/97 showed sensitivity to AgNSs but resistance to AgNWs and AgNRs. In addition, resistance increased with the time of exposure to AgNPs (Fig. 4a).

### Biofilms are affected by AgNPs in a way that depends on both the bacterial species involved and the concentration and shape of the AgNP

AgNPs were able to produce a biocidal effect on biofilms produced by different bacteria, although the results showed different patterns to those obtained for planktonic bacteria. In addition, the effect was variable and depended on various factors.

**Fig. 5:**
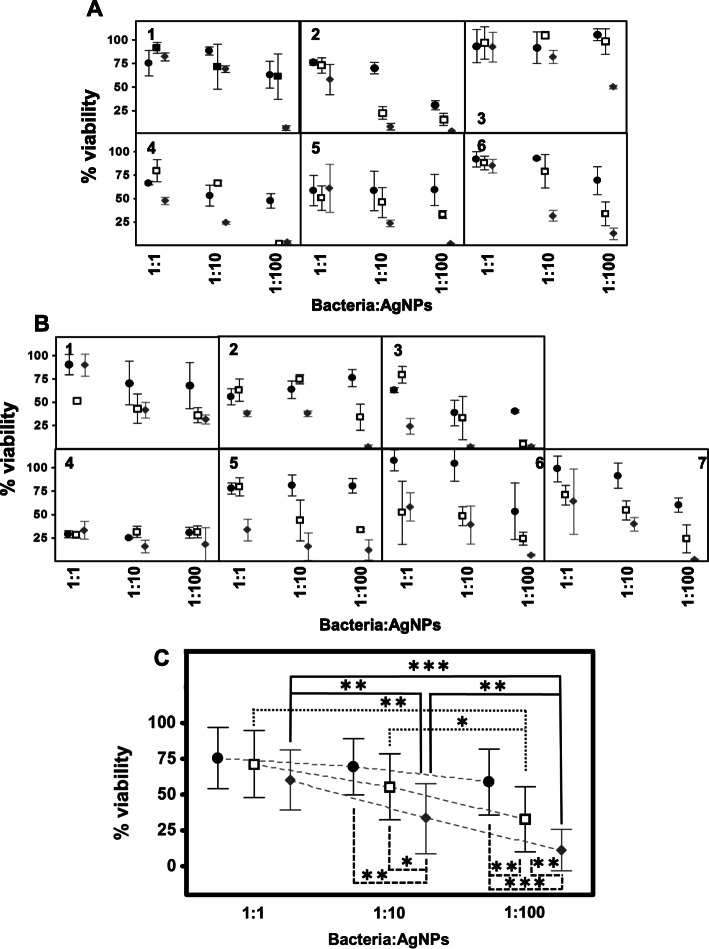
Effect of AgNPs on biofilms. **a** Effect of AgNPs on biofilms generated by Gram positive bacteria: 1, *S.aureus*; 2, *S. epidermidis*; 3, *B. globisporus*; 4, *S. pneumoniae*; 5, *E. faecalis*; 6, *S. pyogenes*. **b** Effect of AgNPs on biofilms generated by Gram negative bacteria: 1, *E. coli;*2, *H. influenzae*; 3, *K. pneumoniae*; 4, *S. typhimurium*; 5, *S. marcescens*; 6, *P. aeruginosa*; 7, *N. gonhorreae*. (●) AgNSs, (□) AgNWs, (♦) AgNRs. **c** Comparison of the effect produced by each specific type of nanostructure on the set of bacteria analyzed using different bacteria:AgNP ratios. Statistically significant differences are denoted by ***, **, and *, which indicate *p* < 0.001, *p* < 0.01, and *p* < 0.05 respectively

First, general patterns were not observed that depended on the Gram positive or Gram negative nature of the microorganism. However, notable differences between the bacterial species analyzed were detected: while some microorganism, such as *B. globisporus*, did not experience any sharp decrease in viability induced by the treatments, the bactericidal effect was strong in other such as *S. pneumoniae*, *E. faecalis*, *S. marcescens* and *N. gonorrhoeae* (Fig. 5a and b).

Both the type of nanostructure and the bacteria:AgNP relationship had a decisive influence on the effects observed. In general, and in contrast to the results obtained for planktonic bacteria, AgNRs were more effective (*S. aureus, S. epidermidis, B. globisporus, E. faecalis, S. pyogenes, H. influenzae, S. marcescens, P. aeruginosa, N. gonorrhoeae*), although in some cases the AgNWs were able to produce similar effects (*S.pneumoniae, E. coli, K. pneumoniae*) (Fig. 5a and b).

The analysis of the pooled results for all the microorganisms studied did not show any significant differences between the different types of AgNPs at bacteria:AgNP ratios of 1:1, although AgNRs showed a greater, and statistically significant, effect at bacteria:AgNR ratios of 1:10 (*p* < 0.05 and *p* < 0.01 compared to AgNWs and AgNSs, respectively), and 1:100 (*p* < 0.01 and *p* < 0.001 compared to AgNWs and AgNSs, respectively). AgNWs, meanwhile, showed toxic effects superior to those of AgNSs, although the difference was only statistically significant at the 1:100 ratio (*p* < 0.01) (Fig. 5c).

Increases in the bacteria:AgNP ratio also caused a decrease in viability in a general way. The results showed statistically significant differences between AgNWs at ratios of 1:100 compared to 1:10 and 1:1 (*p* < 0.05 and *p* < 0.001, respectively), although the comparison between the values obtained at ratios of 1:1 with 1:10 did not reach statistical significance. On the other hand, AgNRs showed statistically significant increases in their antibacterial effect as their ratio to bacteria increased in all the cases studied (Fig. 5c). The differential effect of AgNSs, AgNWs and AgNRs on the viability of bacteria in biofilms at different concentrations also had an impact on the correlation between increasing AgNP:bacteria ratio and the antibacterial activity observed: it was low for AgNSs (r = 0.28), higher for AgNWs (r = 0.56) and higher still for AgNRs (r = 0.7) (Fig. 5c).

### Culture medium conditioned by the presence of AgNPs exhibits antibacterial activity, but the effect is not dependent on the release of Ag ions

**Fig. 6:**
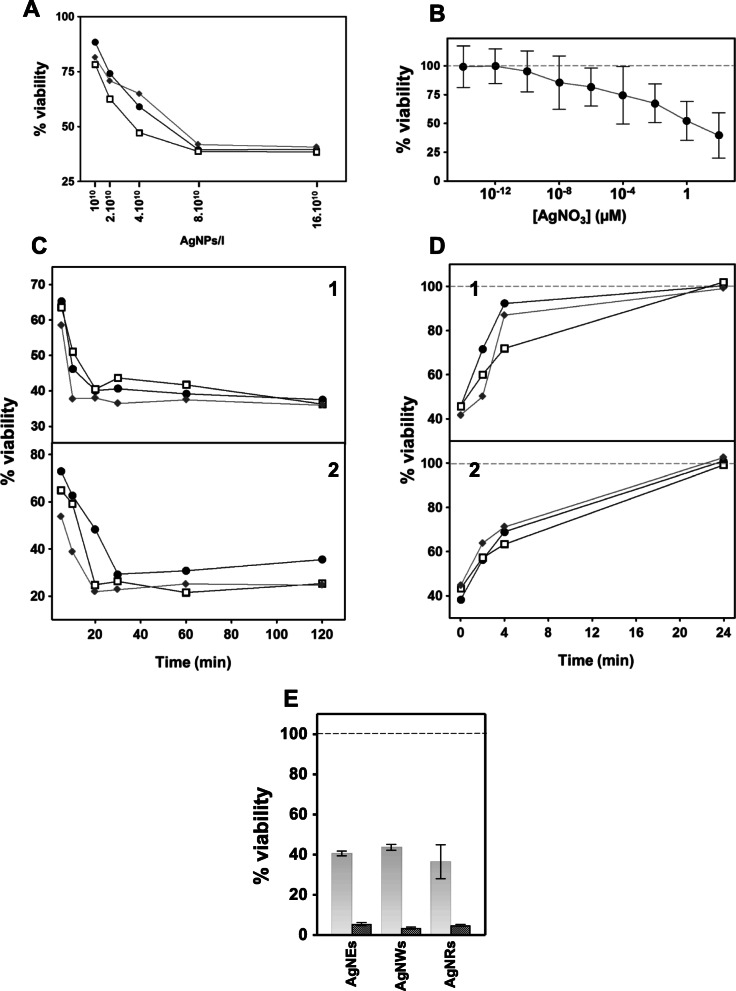
Antibacterial effect of medium conditioned by contact with AgNPs. **a** Effect of BHI conditioned by 5 min of contact with different concentrations of AgNSs, (●), AgNWs, (□) AgNRs (♦) on the viability of *E. coli*. **b** Effect of AgNO3 concentration on the viability of *E. coli* viability**. c** Influence of contact time with AgNPs on the antibacterial activity of BHI (**1**) and H_2_O (**2**) conditioned with AgNSs, (●), AgNWs, (□), AgNRs (♦). **d** Temporal decay of the antibacterial activity of BHI (**1**) and H_2_O (**2**) from the moment of the arrest of its conditioning with AgNSs, (●), AgNWs, (□), AgNRs (♦). **e** Comparative antibacterial effect of AgNPs and BHI conditioned

BHI was conditioned with different concentrations of AgNPs, and the antimicrobial activity of the conditioned medium was evaluated using *E. coli* as a test organism. The results showed the existence of concentration-dependent antibacterial activity (Fig. 6a).

To analyze the possibility that the effect could be due to the release of Ag ions by the nanostructures, the viability of *E. coli* was first analyzed when maintained for 30 min in a wide range of AgNO_3_ concentrations; the results showed that viability was reduced by 50% in these conditions at concentrations of around 1 μM (Fig. 6b). Next, the presence of Ag ions in AgNP-free conditioned medium was analyzed through ICP-AES. The tests were carried out using different combinations of BHI and deionized H_2_O with the different nanostructures, both in the presence and absence of *E. coli*. In all cases, the concentration of Ag was below 5 ppb (Table 1), ruling out the possibility of attributing the observed antibacterial effect to the release of Ag ions.

**Table 1:** Presence of Ag ions in AgNP-free conditioned medium analyzed by inductively coupled plasma atomic emission spectroscopy (ICP-AES)

Sample	[Ag] (ppb)
**H**_**2**_**O milliQ**	< 5
**H**_**2**_**O + AgNSs**	< 5
**H**_**2**_**O + AgNW**_**S**_	< 5
**H**_**2**_**O + AgNRs**	< 5
**H**_**2**_**O + AgNSs +** ***E. coli***	< 5
**H**_**2**_**O + AgNW**_**S**_ **+** ***E. coli***	< 5
**H**_**2**_**O + AgNRs +** ***E. coli***	< 5
**BHI**	< 5
**BHI + AgNSs**	< 5
**BHI + AgNW**_**S**_	< 5
**BHI + AgNRs**	< 5
**BHI + AgNSs +** ***E. coli***	< 5
**BHI + AgNW**_**S**_ **+** ***E. coli***	< 5
**BHI + AgNRs +** ***E. coli***	< 5

The possibility that the antibacterial effect was due to the generation of toxic chemical species in the conditioned media was investigated. Since this type of effect would be expected to be dependent on contact time, both BHI and deionized water were conditioned using different contact times with AgNPs, followed immediately by contact with *E. coli*. The results showed the existence of a time dependent toxic conditioning of both media tested for all types of AgNPs analyzed (Fig. 6c).

Another important aspect of this work was to analyze how long the antibacterial effect of conditioned media lasted. To do so, the bactericidal effect on *E. coli* was determined in BHI and deionized water, both previously conditioned by contact with AgNPs at various intervals after the removal of the nanostructures. The results showed a progressive decrease over time in toxicity, including its total disappearance after prolonged periods (Fig. 6d).

To compare the effect of contact with AgNPs with that produced by the conditioned medium, BHI was conditioned for 30 min with the different AgNPs, and its effect on the viability of *E. coli* was compared with incubations of the bacterium with each AgNP at the same concentration (incubation of 30 min in both cases). The results showed that in all cases the viability reduction was markedly greater when there was physical contact with the AgNPs (Fig. 6e).

## Discussion

The antimicrobial capacity of AgNPs both on bacteria [3, 5, 9, 10, 12, 24] and on other types of microorganisms [17, 24, 25] has been widely established, although there is a variety of experimental evidence as regards the molecular basis of the mechanism(s) responsible [4, 11, 12]. In addition, several studies have described the influence of the structure of AgNPs on the effect observed [26, 27]. In this work, we conduct a study of the antibacterial effect of a recently described type of AgNP: nanoring with 80 nm wire diameter and 12–18 μm (average 14 μm) ring diameter, and analyze its antimicrobial properties on different Gram positive and Gram negative bacteria, both planktonic and biofilm forms, and compare them with AgNSs and AgNWs. The bactericidal effect of different AgNSs and AgNWs has been reported in previous studies. However, the comparison of different studies is difficult because of the different physical and chemical properties of the AgNPs used [4]. For this reason, the comparison carried out in this work is of considerable importance, given that the three types of AgNPs used were obtained in parallel using a common methodology.

Several studies have shown that the effect of AgNPs is dependent on various factors, but particularly on size, shape and concentration [7, 8]. Although the effect of concentration is bacterial dependent, it has been found that complete growth inhibition is achieved at concentrations > 75 μg/ml [11]. To determine the effect on planktonic bacteria, AgNP:bacteria ratios of 1:1 were used in this study, which resulted in variable mortalities of between 59 and 99.3% depending on the bacteria involved, although the median value was 86.1%, with a standard deviation of 10.2%. Taking into account the dimensions of the AgNPs, the concentrations used in these experiments were, respectively, 8.2 × 10^− 3^ μg/μl, 7.4 μg/μl and 0.7 μg/μl for AgNSs, AgNWs and AgNRs, which places them clearly below the values ​​capable of producing the complete inhibition cited in the literature, and thus allowing a better analysis of the toxic effect of the different AgNPs.

In all cases the toxic effect of AgNSs was greater than that of AgNWs or AgNRs on each group of bacteria analyzed, with the exceptions of *N. gonorrhoeae*, where the greatest effect was seen with the AgNRs, and *E. coli*, where the toxicity values ​​of the three different structures were similar. Interestingly, contact time clearly had an influence on the effect: while for AgNSs 5 min contact time was sufficient to reach maximum toxicity values, for AgNRs and AgNWs showed the effect was actually dependent on incubation time in many of the cases. These results clearly reinforce previous findings that the form of AgNPs impacts on their antibacterial capacity, an effect that becomes more evident when we consider that the AgNP:bacteria ratio remained constant in all experiments, although the surface area and volume values ​​are clearly higher for AgNRs and AgNWs when compared to AgNSs (AgNRs having a1400-fold and AgNWs a 6000-fold greater surface area than AgNSs and, respectively, an 85-fold and 900-fold greater volume). These results are in accord with the previously described finding that the reduced size of AgNPs has a favorable influence on their effect [11, 12]. In addition, there is evidence that size and shape influence aspects such as the ability to interact with microorganisms [8], to produce alterations in the cell membrane [28], and to penetrate the bacterial interior [29]. The observable differences between AgNRs and AgNWs were not pronounced, despite their clearly different morphology, and the differences in their respective surface areas and volumes (AgNWs have 4 times the surface area and 10 times the volume of AgNRs).

When the effects produced by AgNPs on Gram positive and Gram negative bacteria were compared, mean survival values ​​were lower in Gram positive bacteria, although the differences were not statistically significant due to the dispersion of the data obtained for Gram negatives. In addition, AgNWs and AgNRs were found to increase their bactericidal effect over time with Gram positive bacteria, resulting in statistically significant correlations, while this was not observed for Gram negatives. These data seem to differ from those obtained in certain previous studies, where Gram positive bacteria were found to be less susceptible to AgNPs than Gram negatives [9, 11], although, other authors have, in contrast, reported a greater internalization of AgNPs in Gram positive bacteria which they conclude is due to their lower surface charge [30].

The mortalities obtained under our experimental conditions were not 100%, and so the growth of the surviving fraction of microorganisms in long incubation periods was analyzed. It was found that in these circumstances, the efficiency of the different types of AgNPs differed to that observed over short time periods. AgNSs reduced the rate of bacterial growth to differing extents depending on the bacterial species involved, while AgNWs displayed a stronger inhibitory effect with all the bacteria studied, and AgNRs, were able to produce the practically total inhibition of growth even at the lowest concentrations. These results do not support the notion that, in prolonged incubation periods, the reduced size of the AgNSs favors its effect. Nor do they support the idea that increased toxicity could be the result of mechanisms such as the possible release of Ag ions because, as indicated above, AgNWs have a volume 10 times that of AgNRs and a surface area that is 4 times greater. These observations may point to the shape of the particle being an important element in the effectiveness of the AgNP. Other possible factors could be the physiology of the microbial fraction that is capable of surviving treatment with AgNPs, which, in turn, might be linked to intrinsic resistance mechanisms such as gene deregulation, membrane permeability or the existence of persistent populations, or with possible mechanisms of altruistic behavior based on programmed cell death [18, 31, 32].

The use of AgNPs in medical applications is of great interest due to the rapid development and spread of antibiotic resistances. However, in different microorganisms such as *E. coli*, *P. aeruginosa* and *Bacillus sp*., the appearance of resistance to AgNPs has been reported after repeated exposure to these structures [18, 33]. *S. typhimurium* is a particularly interesting case, since it was the first clinical bacterium in which resistance mechanisms were described [31] and, in addition, the effect was serovar dependent [34]. In this study, the resistance to different types of AgNSs of two Ag resistant *Salmonella* strains, *S. enterica* 207/07 and *S. enterica* 389/97, were analyzed. The results showed two clearly differentiated patterns, with high resistance to AgNSs in strain 207/07, while 389/97 was very sensitive to AgNSs and, in contrast, resistant to AgNRs and, particularly, to AgNWs. These results reinforce the finding that the three morphologies act differently, the response of serovars being dependent on the intrinsic and extrinsic mechanisms they possess, which may include adaptive mutations or plasmids carrying resistance genes [34, 35].

In numerous medical and biotechnological applications, bacteria do not appear in their planktonic form, but rather adhere to surfaces and form biofilms. In biofilms, bacteria show specific biological properties, which include differential gene expression, physiological modifications or the presence of an extracellular matrix, all of which clearly modify their response to antimicrobial agents [32]. It has been described that AgNPs can exert anti-biofilm activity, although fewer studies focus on this than on planktonic bacteria, and the mechanism of the action of AgNPs on these structures is not accurately known [4]. In the present study, it was possible to determine that each of the different AgNPs had an anti-biofilm effect, although this followed a different pattern to the effects observed in planktonic bacteria, with AgNRs being the most active structures, and AgNSs the least, which points to the mechanism of the action being different to that involved in the planktonic bacteria. AgNR inhibition data are also important, considering that the median survival of bacteria at a concentration equivalent to 50 pM (1:100 ratio) was 6.4%, whereas other studies in the literature describe survival values of 0.03% for *Mycobacterium smegmatis* at 100 μM [20] or 65% for *P. aeruginosa* and 88% for *S. aureus* at concentrations of 1–2 mM [21]; A study has also recently been published in which biogenic AgNPs obtained by a non-physical-chemical procedure produced the concentration-dependent inhibition of biofilm formation in different bacterial species, including Gram positive and Gram negative bacteria [36].

Although all the fundamental details of the antibacterial action of AgNPs are not known in detail, it is known that they can interact simultaneously with multiple targets in the target cells [31]. Among the mechanical bases of the antimicrobial activity of AgNPs are adhesion to cell covers, modulation of signal transduction pathways, penetration into cells followed by destabilization of molecules, or induction of oxidative stress [11]. Some of these mechanisms do not necessarily involve physical contact with the microorganism and, in fact, it has been widely proposed, as mentioned above, that the release of Ag ions by AgNPs might be implicated in toxicity [11]. The data presented in this article show that when the culture medium is conditioned by contact with AgNPs it displays antibacterial properties without the nanoparticles needing to actually be present. At least in short incubation periods, the effect, therefore, does not appear to be due to the release of Ag ions, since a high enough concentration of ions in the medium was not detected under these conditions. The effect occurs not only in culture medium, but also in conditioned deionized water, where it is dependent on the concentration of AgNPs used and the incubation time. Furthermore, the toxic capacity of the conditioned medium or water disappears progressively in relatively short periods of time. All these data suggest the possible formation of reactive species capable of inducing oxidative stress that could be responsible for, at least part of, the observed effect. However, the result obtained with direct incubation in the presence of AgNPs is greater than that obtained with conditioned media, which suggests that the mechanism of action is mixed, and also involves contact with the microorganism. This mixed mechanism of action seems general to the 3 types of AgNPs tested in the present study.

## Conclusions

In conclusion, in this work we test the antibacterial capacity of a new type of previously described nanomaterial, AgNRs, and compare it with other nanostructures, AgNS and AgNWs, obtained by the same procedure. AgNRs show an antibacterial capacity on planktonic microorganisms that is lower than that of AgNSs over short contact periods, although for longer periods they are able to inhibit bacterial growth more efficiently. In addition, AgNRs show greater effectiveness than other nanostructures against biofilms. The mechanism of resistance in Salmonella serotypes resistant to Ag shows differences depending on the strain involved, suggesting that different AgNPs can act through different mechanisms, and that these mechanisms are probably based, at least in part, on the conditioning of the environment by the AgNP involved through the formation of toxic chemical species.

## Methods

### Bacterial strains and cell lines

The bacterial species used in this study were *Staphylococcus aureus*, *Staphylococcus epidermidis*, *Streptococcus pyogenes*, *Streptococcus pneumoniae*, *Enterococcus faecalis*, *Bacillus globisporus*, *Escherichia coli*, *Serratia marcescens*, *Haemophilus influenzae*, *Klebsiella pneumoniae*, *Neisseria gonorrhoeae* and *Pseudomonas aeruginosa* (clinical isolates obtained from the Hospital Universitario Central de Asturias) and silver resistant strains *Salmonella enterica* serovar *typhimurium* 389/97 (LSP 389/97) [37] and *Salmonella enterica* serovar *typhimurium* 207/07 (LSP 207/07) (obtained from the laboratory of Dr. Rosario Rodicio at the University of Oviedo).

All the bacteria were grown in Brain Heart Infusion (BHI) broth (Gibco-Thermo Fischer Scientific, Waltham, MA, USA) except *H. Influenzae*, which was grown in BHI including *Haemophilus* Test Medium Supplement (Gibco). All the bacteria were grown at 37 °C in a shaking incubator, except *S. pneumoniae*, *N. gonorrhoeae* and *H. influenzae,* which were grown in a 5% (v/v) CO_2_ atmosphere without shaking.

### Synthesis of silver nanostructures

The synthesis of AgNPs was carried out using a modified polyol-mediated process [22, 23]. This involved the reduction of AgNO_3_ (Sigma-Aldrich, St. Louis, USA) in ethylene glycol (EG) (Panreac, Barcelona, Spain) in the presence of polyvinylpyrrolidone 360 k (PVP) (Sigma-Aldrich). In the first step, 0.27 mg of CuCl_2_:2H_2_O (Panreac) was added to 50 ml of EG followed by the simultaneous addition of a solution of AgNO_3_ (240 mg of AgNO_3_ in 15 ml of EG) and a solution of PVP (190 mg of PVP in 15 ml of EG) using a peristaltic pump, at a rate of 1.5 ml/min for 20 min. At the end of this process the solution turned from a transparent to a pearly appearance, denoting the presence of silver nanostructures and, after 5 min, the agitation was stopped for another 15 min. The reaction was performed at 170 °C (and always below 175 °C) under magnetic stirring. Once the reaction was completed, the flask was quickly submerged in ice water until the contents reached room temperature.

Anhydrous ethanol and pure acetone were used in the separation and purification steps, and all chemical reagents and solvents were of analytical grade and used without further purification. In brief, AgNSs were separated by centrifugation (4000–1000 rpm) from the reaction medium and resuspended for purification. The remaining suspension was left to decant for 3 days, after which a concentrated solution of AgNRs, together with a small amount of AgNWs, were obtained from the supernatant of this suspension, while the remaining suspension provided the majority of the AgNWs. The AgNPs were suspended in water, at a suitable concentration for the subsequent processing. The structural features of the nanostructures obtained through this process were: AgNSs, 40–60 nm in diameter; AgNRs, 80 nm wire diameter, 12–18 μm (average 14 μm) ring diameter; AgNWs, 200 nm in diameter and 50–100 μm in length. FEG-SEM Images of AgNPs, AgNWs and AgNRs are shown in Figure S1 (Additional file 1). To ensure the equivalence between differente batchs, the corresponding analyses (FEG-SEM, UV-spectrophotometry, ATG-ATD) were carried out after each synthesis.

In order to check the initial concentration of the AgNPs, a gravimetric method was used. A cotton swab was heated overnight, at 110 °C to a constant weight (in triplicate); 1 ml of suspension was added to another cold cotton swab and then heated at 110 °C for 4 h, until the weight remained constant. Calculating the difference in weight between the two swabs enabled the content of nanostructures, measured in grams per millilitre to be obtained. The final concentrations were: 1.7 × 10^7^ AgNSs /μl suspension; 6.0 × 10^4^ AgNRs /μl suspension; 2.4 × 10^4^ AgNWs /μl of suspension.

### Effect of AgNPs on cell viability in planktonic bacterial cultures

To analyze the bactericidal effect of the different AgNPs, bacterial cultures grown at an A_600_ of 0.5 were kept at room temperature and in agitation with the nanomaterials for periods of time between 5 and 30 min. Next, the nanostructures were removed by centrifugation at 800 rpm for 2 min, and different dilutions of the supernatant were seeded on solid BHI medium plates. After incubation of the plates at 37 °C overnight, the colonies obtained were quantified.

The bacterium:nanostructure ratio used as standard in the tests had been previously estimated from cultures of *E. coli* maintained for 30 min under agitation with AgNWs in the following ratios (bacteria:AgNWs): 10^8^:1, 10^6^:1, 10^4^:1, 10^2^:1, 1:1, 1: 10, 1: 10^2^, 1: 10^3^, 1: 10^4^. The 1: 1 ratio was chosen as the most suitable for the assays

### Effect of AgNPs on cell growth in planktonic bacterial cultures

Their influence on bacterial growth was analyzed by incubating the different AgNPs with bacterial cultures at an A_600_ of 0.02, and using bacteria:nanostructure proportions of 1:1, 1:2 and 1:3. The effect was quantified by determining absorbance after incubation periods of 1, 2, 3 and 4 h.

### Effect of AgNPs on bacterial biofilms

To analyze the effect of AgNPs on biofilms, bacterial cultures at an A_600_ of 0.01 were added to 96 well plates (100 μl/well) which were incubated for 24 h. AgNPs of different morphologies were then added in the proportions (bacteria:AgNP) 1:1, 1:10 and 1:100. The plates were incubated again for 24 h at 37 °C. Biofilms were treated with PBS with 0.25% triton X100 for 1 min, and different dilutions of these extracts were seeded onto solid BHI medium plates and, after incubation at 37 °C overnight, the colonies obtained were quantified.

### Effect of AgNO_3_ on *E. coli* viability

*E. coli* cultures at an A_600_ of 0.5 were treated with AgNO_3_ at concentrations of 10^2^ μM, 1 μM, 10^− 2^ μM, 10^− 4^ μM, 10^− 6^ μM, 10^− 8^ μM, 10^− 10^ μM, 10^− 12^ μM, 10^− 14^ μM and for 30 min at 37 °C with stirring. After this time, aliquots were inoculated in solid BHI medium, the plates were incubated overnight, and the colonies obtained were quantified.

### Determination of the concentration of silver ions released by AgNPs

Different samples of *E. coli* cultures in BHI at an A_600_ of 0.5, sterile BHI, suspensions of *E. coli* in deionized water at an A_600_ of 0.5 or sterile deionized water were individually treated with amounts of AgNSs, AgNWs and AgNRs equivalent to a bacteria:AgNP ratio of 1:1 in all cases. After incubating for 30 min with stirring, the suspensions were centrifuged at 3000 rpm for 5 min to discard the AgNPs. The concentration of Ag in the particle-free supernatants was determined by inductively coupled plasma atomic emission spectroscopy (ICP-AES) using an Agilent 5110 ICP-OES Instrument (Agilent, Santa Clara, CA, USA).

### Study of the toxic effect of media conditioned by AgNPs

To analyze the effect that conditioning through the presence of AgNPs might have on bacterial viability, aliquots of deionized water or BHI were kept in contact with nanostructures at different concentrations (10^10^, 2 × 10^10^, 4 × 10^10^, 8 × 10^10^, 16 × 10^10^ Units/l) for a variable time (5, 10, 20, 30, 60 or 120 min, depending on the experiment) with stirring. Next, the AgNPs were removed by centrifugation at 3000 rpm for 5 min, and *E. coli* was incubated in the conditioned media at an A_600_ of 0.5 for 30 min with stirring. The amount of nanostructures used in the experiments was adjusted to a bacteria:AgNPs ratio of 1:1, equivalent to that used in the studies of cell viability in planktonic bacterial cultures. Finally, different dilutions of these cultures were seeded onto solid BHI medium plates and, after incubation at 37 °C overnight, the colonies obtained were quantified.

In order to establish the temporary durability of the conditioning of the culture medium, aliquots of deionized water and BHI were kept in contact with AgNPs at the same concentrations described in the previous paragraph, after which the AgNPs were removed by centrifugation at 3000 rpm for 5 min, and the supernatant reserved at room temperature for 0, 2, 4 and 24 h. Each sample of conditioned medium was incubated with *E. coli* at an A_600_ of 0.5 for 30 min with stirring, and the quantification was carried out by dilution in solid BHI medium as described above.

### Statistical analysis

The results were analyzed using a Kruskal-Wallis test in the Statistics for Windows program (Statsoft Inc.; Tulsa, OK). The differences were considered significant when *p* < 0.05.

## Supplementary information

**Additional file 1: Figure S1.** FEG-SEM Images of Silver nanoparticles.

## Data Availability

The data sets used and/or analysed during the current study are available from the corresponding authors on reasonable request.

## Abbreviations

AgNPs: Silver nanoparticles
AgNRs: Silver nanorings
AgNSs: Silver nanospheres
AgNWs: Silver nanowires
EG: Ethylene glycol
ICP-AES: Inductively coupled plasma atomic emission spectroscopy
NPs: Nanoparticles
PVP: Polyvinylpyrrolidone
