# Supplementary material for: Antibacterial effect of silver nanorings
Source: BMC Microbiol. 2020 Jun 19;20:172. doi: 10.1186/s12866-020-01854-z (PMC7304143; doi:10.1186/s12866-020-01854-z)
Supplement: Supplementary file 1 — Additional file 1: Figure S1. FEG-SEM Images of Silver nanoparticles. [file 12866_2020_1854_MOESM1_ESM.pdf]

Figure S1. FEG-SEM Images of Silver nanoparticles

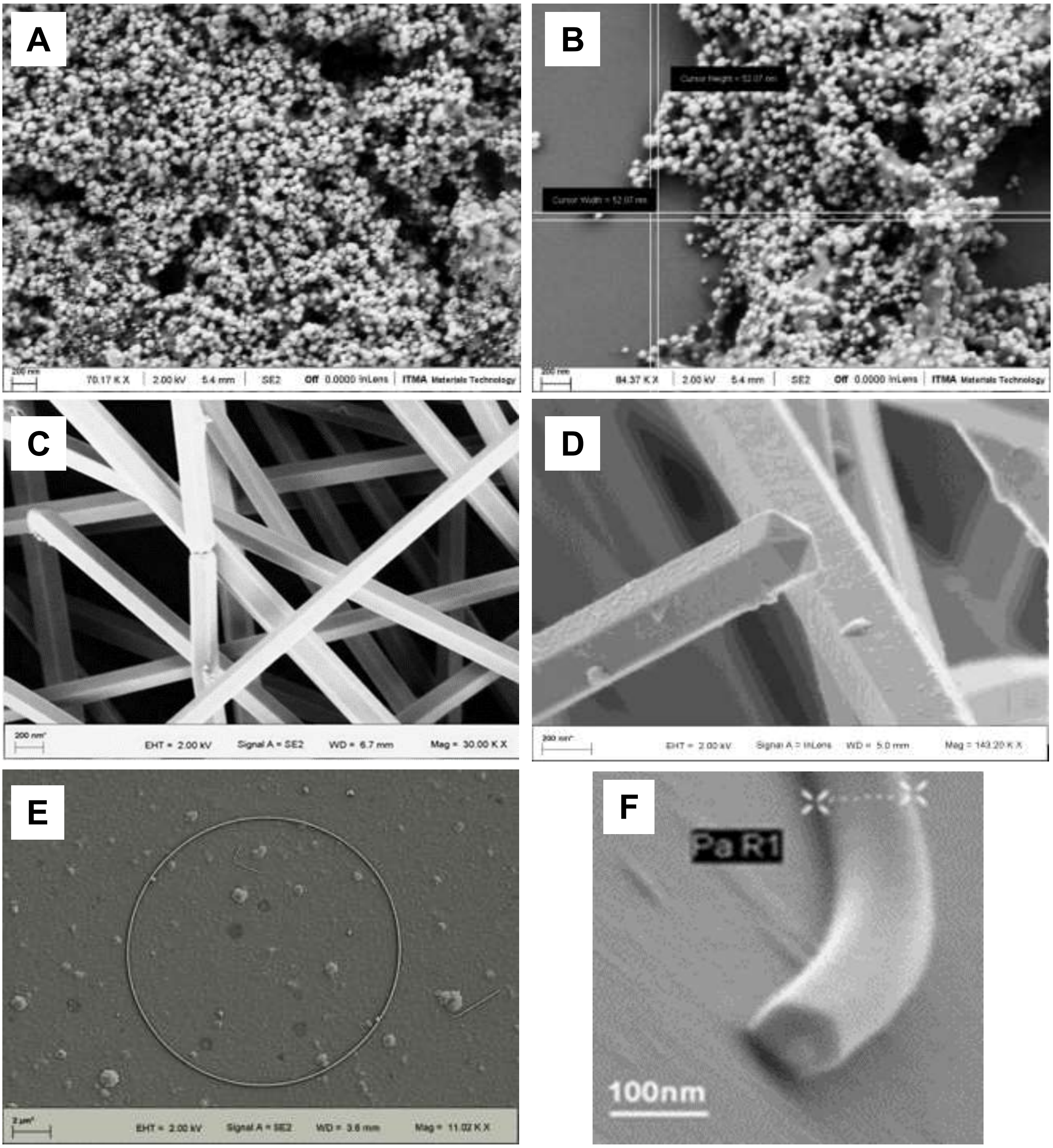

A, B: FEG-SEM Images of silver nanospheres; **C,D**: FEG-SEM Images of silver nanowires;
E,F: FEG-SEM Images of silver nanorings
